# Vitamin D Deficiency in Pediatric Fracture Patients: Prevalence, Risk Factors, and Vitamin D Supplementation

**DOI:** 10.4274/jcrpe.3474

**Published:** 2016-12-01

**Authors:** Erwin A. Gorter, Wilma Oostdijk, Abraham Felius, Pieta Krijnen, Inger B. Schipper

**Affiliations:** 1 Leiden University Medical Center, Department of Surgery and Traumatology, Leiden, The Netherlands; 2 Leiden University Medical Center, Department of Pediatrics, Leiden, The Netherlands

**Keywords:** Vitamin D, Vitamin D deficiency, child, infant, fracture, fracture healing

## Abstract

**Objective:**

Although vitamin D levels are not routinely monitored in pediatric fracture patients, identification of children with a vitamin D deficiency may be clinically relevant because of the potential role of vitamin D in fracture healing. This study aimed to determine the prevalence of vitamin D deficiency in a pediatric fracture population and to identify risk factors for deficiency.

**Methods:**

All pediatric patients (<18 years) who were treated for a fracture of the upper or lower extremity from September 2012 to October 2013 in the outpatient setting of a level one trauma center were included in this cross-sectional study. Vitamin D deficiency was defined as a serum calcidiol <50 nmol/L. Potential risk factors for vitamin D deficiency were analysed using multivariable logistic regression analysis.

**Results:**

A total of 108 boys (58%) and 79 girls, of a mean age 11.1 years (standard deviation 3.9), who had undergone 189 fractures were included in the study. Sixty-four children (34%) were vitamin D deficient. Of those with follow-up measurements, 74% were no longer deficient after supplementation. Vitamin D status did not influence the occurrence of complications during fracture treatment. Independent risk factors for vitamin D deficiency were older age, season (spring), and a non-Caucasian skin type.

**Conclusion:**

Clinicians who treat children with a fracture should inform patients and parents on vitamin D supplementation. Vitamin D measurement and supplementation may be needed for children with a non-Caucasian skin type or for those who present with a fracture during spring months.

**WHAT IS ALREADY KNOWN ON THIS TOPIC?**

Vitamin D plays a role in the complex cellular processes of fracture healing, studies that address risk factors for vitamin D deficiency, the clinical effects of vitamin D deficiency or supplementation on fracture healing are scarce and inconclusive.

**WHAT THIS STUDY ADDS?**

Vitamin D deficiency is relatively common in a pediatric fracture population. For children with a non-Caucasian skin type or presentation with a fracture during spring, clinicians might consider vitamin D measurement and supplementation.

## INTRODUCTION

Up to 60% of boys and 40% of girls undergo a fracture during childhood (1,2,3,4,5). Increased participation in competitive sports and the relative under-mineralization of the skeleton during the early phase of the pubertal growth spurt may contribute to the high fracture rate (6). Vitamin D deficiency is considered a global health problem (7). The prevalence in healthy European children varies between 8% and 95% depending on risk factors such as geographical location, sun exposure, skin type, vitamin D supplementation, or the presence of obesity (7,8). Vitamin D is essential for bone mineralization and maintenance of bone quality through its vital role in the regulation of calcium and skeletal homeostasis (9). Although vitamin D plays a role in the complex cellular processes of fracture healing, studies that address risk factors for vitamin D deficiency, the clinical effects of vitamin D deficiency, or the question of supplementation on fracture healing are scarce and inconclusive (9,10).

Low bone mineral density is a risk factor for fractures (11). Infants with severe vitamin D deficiency, such as is present in rickets, have a tendency towards increased fracture rates (12,13). The possible relationship between vitamin D deficiency and the occurrence of fractures in pediatric ages has not yet been established (14,15,16,17). A recent study showed that a lower vitamin D status is associated with fractures requiring surgery, but not with the occurrence of fractures (18). Although the prevalence of vitamin D deficiency in children in the general population has been well described (7,8), the prevalence of vitamin D deficiency in the pediatric fracture population is less often reported with a wide variation ranging from 8% to 47% (Table 1) (14,15,17,18,19,20,21).

The primary aim of the present study was to determine the prevalence of vitamin D deficiency in a pediatric population who had undergone a fracture in the upper or lower extremity. The second aim was to identify the risk factors for vitamin D deficiency in this patient group.

## METHODS

Approval for this cross-sectional study was obtained from the Medical Ethics Review Committee of our institution (P12.058). The study included all consecutive pediatric patients (age <18 years) who were treated for a fracture of the upper or lower extremity between 1 September 2012 and 1 October 2013 in the outpatient clinic of our level 1 trauma center. According to Dutch law, children aged 16 years or older are considered able to give informed consent themselves for study participation. For children of 12 to 16 years consent from both the child and the parents is needed before inclusion. In children younger than 12 years only consent of the parents is necessary. In this study, conservatively treated children and/or their parents received study information and were asked to provide written informed consent in the plaster cast room approximately one week after the fracture. In cases who required surgery, children and/or their parents were asked to provide written informed consent before the intervention.

A blood sample was taken during the first follow-up visit after the fracture incident. The serum concentration of 25-hydroxyvitamin D was measured using an Electro Chemo Luminescence Immuno Assay from Roche Diagnostics (Modular E170). In the literature, there is no consensus on the adequate vitamin D levels and this may explain the inconsistency of reported data related to the effect of vitamin D deficiency on occurrence of hyperparathyroidism, metabolic bone disease, and hypocalcaemia. The American Academy of Pediatrics, the Pediatric Endocrine Society, and the Institute of Medicine all consider a serum concentration vitamin D >50 nmol as sufficient/normal (22,23,24). Also, according to their recommendations, a minimum serum concentration of 50 nmol/L should also be maintained or should be the target value in case of supplementation. Serum concentrations below 50 nmol/L were defined as deficient by the Endocrine Society Clinical Practice Guidelines (25). Based on these definitions and target values, we defined vitamin D deficiency as a serum 25-hydroxyvitamin D level <50 nmol/L (20 ng/mL) in our study. Patients with low vitamin D levels were referred to a pediatrician for further assessment, supplementation according to the schedule presented in Table 2, and follow-up.

Body mass index (BMI) was determined according to gender and age. Classification of being underweight, having a normal weight, or being overweight or obese was based on the BMI distribution for Dutch boys and girls in 2009 (26). Month of fracture was categorized into autumn (September, October, November), winter (December, January, February), spring (March, April, May), and summer (June, July, August) months.

The children and/or their parents completed a questionnaire on potential risk factors for vitamin D deficiency including medical history, medication, sun exposure, and vitamin D usage prior to the fracture (27). In the questionnaire, daily UV-radiation exposure was defined as the average number of hours spent outdoors between 10:00 am and 03:00 pm (27,28,29). Skin type was determined using the Fitzpatrick scale (30). According to this scale, individuals are classified as type 1: pale white skin, always burns, never tans; type 2: white skin, burns easily, tans minimally; type 3: white skin, burns moderately, tans uniformly; type 4: light brown/moderate brown skin, burns minimally, always tans well; type 5: brown skin, rarely burns, tans profusely; type 6: dark brown to black skin, never burns.

Complications concerning fracture healing were documented as follows: refracture, epiphysiodesis, malunion, delayed union, and non-union which occurred within 6 months after the fracture.

### Statistical Analysis

Patient characteristics are presented as means and standard deviations (SD) or as percentages. Patient groups were compared using the student’s t-test for continuous variables and the chi-squared test or Fisher’s exact test for categorical data, as appropriate. Patient characteristics with a univariable association (p≤0.10) with vitamin D deficiency were combined in a multivariable logistic regression analysis to identify independent risk factors for these conditions. The strength of selected risk factors was expressed as the adjusted odds ratio (OR) with its corresponding 95% confidence interval (CI). Statistical analysis was performed with Statistical Package for the Social Sciences software version 20 (IBM SPSS Statistics for Windows, Version 20.0. Armonk, NY: IBM Corp.). A p-value of <0.05 was considered statistically significant.

## RESULTS

**Figure 1:**
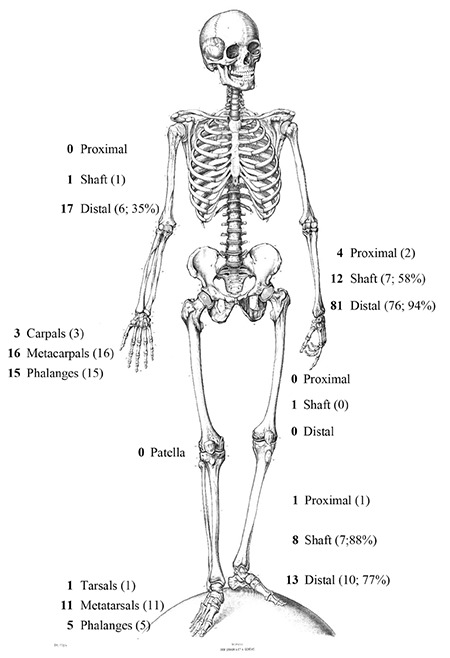
Fracture location and treatment of 189 fractures. The bold numbers indicate the number of fractures. The number (%) of conservatively treated fractures are indicated between parentheses

A total of 587 children with fractures (40% located in the distal forearm) were found to be eligible for the study. Of these, 352 (60%) were boys and 235 were girls. The mean age of the group was 10.2±4.1 years. Of these children, 187 (31.9%) agreed to participate in the study and provided written informed consent. In the study group, 108 were boys (58%) and 79 girls. The mean age of the group was 11.1±3.9 years (Table 3). Together they sustained 189 fractures, of which, the most frequent (43%) were distal forearm fractures (Figure 1). Most of the fractures were treated conservatively (n=161; 85%). The majority of the fractures which required surgery were treated with K-wires (14/28) or Elastic Stable Intramedullary Nailing (6/28). Of the 187 children, 73 (39%) had previously sustained a fracture (Table 2).

The number of patients receiving medication was 23 (18%). These medications were predominantly antiallergic drugs (salbutamol and/or salmeterol /fluticasone) and drugs for attention-deficit hyperactivity (methylphenidate) or diabetes (insulin). Most patients (n=163, 88%), had a Caucasian skin type (Fitzpatrick skin type 1, 2, or 3). Vitamin D supplements were used by 24 (13%) patients mostly as a component in a multivitamin preparation. Although recommended in the Netherlands, only 4 of the 5 children younger than four years were on vitamin D supplements, and only one of the 22 children with a dark skin type (4 or 5) aged 4 years and older was receiving vitamin D supplementation.

The blood sample for determination of serum 25-hydroxyvitamin D levels was taken at a median time of 8 days after the fracture (range 0-69 days). With a mean of 64.9 (SD 27), a total of 123 children (66%) had a 25-hydroxyvitamin D level ≥50 nmol/L and 64 children (34%) were vitamin D deficient (25-hydroxyvitamin D <50 nmol/L).

Potential risk factors (univariable p≤0.10) for vitamin D deficiency were higher age, non-Caucasian skin type, and season (winter and spring) (Table 3). In the older age groups we found more vitamin D deficiency: 16% for children younger than 10 years, 46% in age group 10-16 years and 41% in children aged 16-18 years (p=0.001). A potentially protective factor against vitamin D deficiency was a holiday with high sun exposure within the previous month. Combined in the multivariable logistic regression model, all these factors were shown to be independent risk/protective factors for vitamin D deficiency.

**Figure 2:**
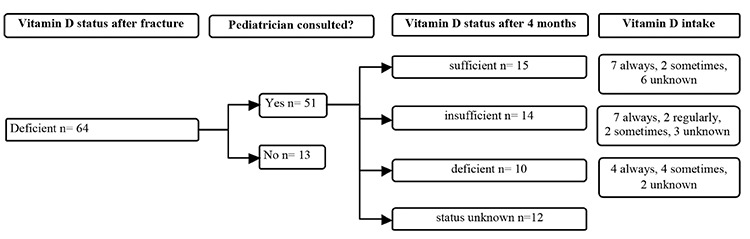
Flow chart of follow up of children with a vitamin D deficiency, vitamin D supplementation and results after supplementation

Of 64 children with vitamin D deficiency who were referred to the pediatrician, 51 actually visited the pediatrician (Figure 2). No clinical, biochemical, or radiological signs of rickets were found in any of these children. Osteopenia was diagnosed with a dual-energy X-ray absorptiometry scan in one of the two children with celiac disease. All 51 children were treated according to the protocol shown in Table 1. In 39 of them, the serum 25-hydroxyvitamin D measurement was repeated after 4 months; 29 (74%) were no longer vitamin D deficient (Figure 2). No vitamin D intoxication occurred in any of the supplemented children.

The mean follow-up period in the 160 conservatively treated patients was 6.1 weeks (range 1-59 weeks). During the cast immobilization which lasted on average 3.7 weeks, no complications occurred. In 3 of the 160 children, a refracture occurred respectively within one month, after 6 weeks, and after 5 months after removal of the cast. In these 3 children, the initial 25-hydroxyvitamin D levels were 119, 39, and 23 nmol/L, respectively. Only in the last patient, the vitamin D level was determined at the second presentation and was shown to have reached a sufficient level. The occurrence of complications after cast immobilization was not related to the initial vitamin D status in this cohort. The 28 children with a surgically treated fracture had an average follow-up of 15.4 weeks (range 1-42 weeks). In 21 children, the fixation material was removed according to the treatment protocol. In the surgically treated group, all fractures healed without complications within 6 months after treatment.

## DISCUSSION

The results of this study show that 34% of the pediatric fracture patients had vitamin D deficiency. However, no patient had the clinical signs of rickets. Higher age, a non-Caucasian skin type, and spring season were independent risk factors for vitamin D deficiency. After four months of treatment with vitamin D, 74% of the children who initially had vitamin D deficiency were no longer vitamin D deficient.

In the literature, the prevalence of vitamin D deficiency in the pediatric fracture population is reported to be between 8% and 47% (Table 1). Inclusion to our study was not limited to certain age groups, type of treatment, skin type, or fracture location. Therefore, the observed frequency of 34% for vitamin D deficiency probably also reflects the prevalence in the general pediatric fracture population. Schilling et al (15) found a far lower incidence of 8% vitamin D deficiency in 118 children younger than 2 years with a fracture. This low prevalence may be age- and country-dependent due to the recommendation of the American Academy of Pediatrics to supplement vitamin D in the very young children (31). As no children younger than 2 years were present in our series, we could not compare these data with our results. Ryan et al (14) examined 76 African-American children with a forearm fracture and found 47% to be vitamin D deficient. The inclusion of only children with a dark skin type, a risk factor for having a vitamin D deficiency, probably explains why they found so many more vitamin D deficient children in their population compared to our pediatric population. This obvious variation in vitamin D deficiency prevalence clearly reflects the presence or absence of certain risk factors. The five children with a dark skin type (type 5 or 6) who were included in our study were indeed all vitamin D deficient. Olney et al (21) retrospectively identified children with a history of two or more fractures and found a vitamin D deficiency prevalence of 21% in this group. The results of James et al (19) were limited to children with an upper extremity fracture and showed a vitamin D deficiency in 24%. Ceroni et al (20) included 100 adolescent (between 10 and 16 years) patients with upper- or lower-limb fractures and found that 12 (12%) were vitamin D deficient. We documented a prevalence of 46% vitamin D deficiency in 98 children between 10 and 16 years. Ceroni et al (20) only included surgically treated children in their series and measured the vitamin D concentration at once after storage, which may explain the difference in prevalence. Similar to our study, Contreras et al (17) did not limit inclusion to the study to age or fracture location, although they did not report the patients’ skin type. Minkowitz et al (18) included all fracture locations in a population aged between 2 and 18 years. These two authors, respectively, reported vitamin D deficiency in 20% and 18% of their subjects. The seeming differences in prevalence between our and other studies may have resulted from seasonal differences and differences in geographical distribution or latitude (27,32), but also from differences in characteristics of the study populations. It should be noted that in our study, only 12% of the children had a non-Caucasian skin type and our study population tended to be more close to adolescence with a mean age of 11.1 years.

To our knowledge, only James et al (19) and Minkowitz et al (18) described risk factors for vitamin D deficiency in a pediatric fracture population. Although not tested in a multivariable analysis, they also described a significant effect of skin type on the serum concentration of vitamin D. In contrast to our results, age and season did not seem to affect the serum calcidiol level in the study of James et al (19). Contreras et al (17) merged the fracture group with the non-fracture group and described risk factors for an insufficient vitamin D concentration (<75 nmol/L). They also found a higher prevalence of insufficiency in non-Caucasian children, as well as in children presenting in the winter and spring. Some established risk factors for vitamin D deficiency in a nonfracture population have been reported in the literature. One of these is obesity (22). Because vitamin D is a fat-soluble vitamin, a higher dose of vitamin D supplementation in obese children is recommended (22). Although some studies have identified obesity as a risk factor for vitamin D deficiency, we did not find this relation in our pediatric fracture population.

Results of studies in many countries and also national data on Dutch children indicate that prevalence of vitamin D deficiency is not expected to differ in children with or without a fracture (14,17,18,20,21,33,34). Thus, routine vitamin D measurement in children with a fracture should be avoided. The prevailing advice of the National Health Councils also render routine vitamin D measurement in children with a non-Caucasian skin type unnecessary. The Dutch Health Council advises daily vitamin D supplementation of 400 IU in all children up to four years in order to prevent rickets (35). In children of four years and older, the Health Council advises standard daily vitamin D supplementation with 400 IU in children with a light skin type (Fitzpatrick skin type 1, 2, or 3) who have insufficient daily sun exposure (<15 min between 11:00 am and 03:00 pm) and in all children with a dark skin type (Fitzpatrick skin type 4, 5, or 6) (35). This dose is consistent with estimated average requirement described by the Institute of Medicine (24). The Endocrine Society Clinical Practice Guideline recommends a higher (600-1000 IU) daily dose for children at risk for vitamin D deficiency. Our results indicate that these recommendations are poorly implemented; only 1/22 children with a dark skin type aged ≥4 years had received vitamin D supplementation prior to the study. And we identified a non-Caucasian skin type as an independent risk factor for vitamin D deficiency, a result also reported by others (17,18,19). The overall awareness of the importance of an adequate vitamin D status in these children and knowledge of the advice of the Health Council (supplementation with 400 IU per day in the risk population) should become part of the fracture treatment protocol.

A limitation of our study was the low participation rate. The most commonly stated reasons given by children and their parents for non-participation were fear of blood collection and increased time spent in the hospital. The low participation rate may have introduced a selection bias, but the included group of children seemed representative based on the available information of all eligible children regarding age, gender, fracture location, and seasonal distribution. Although blood samples were obtained as soon as possible after the fracture had occurred, this took place up to two months after initial trauma and this delay in some patients may have resulted in a less accurate information on the vitamin D status at the time of injury. Another limitation was that data concerning fracture healing were obtained retrospectively, with all well-known shortcomings of retrospective data acquisition.

In conclusion, this study has shown that one in three children with a fracture can be vitamin D deficient. Nevertheless, routine vitamin D measurement in children with fractures is not recommended. The results of our study also show that higher age, a non-Caucasian skin type, and spring season are risk factors for vitamin D deficiency in pediatric fracture patients. Clinicians who treat children with a fracture should inform the patient and their parents about the prevailing advice regarding vitamin D supplementation and also note the presence of potential risk factors. Vitamin D measurement and supplementation can be considered in children with a non-Caucasian skin type and in those who present with a fracture during spring months.

## Ethics

Ethics Committee Approval: Approval for this cross-sectional study was obtained from the Medical Ethics Review Committee of our institution (P12.058), Informed Consent: It was taken.

Peer-review: Externally peer-reviewed.

## Figures and Tables

**Table 1:**
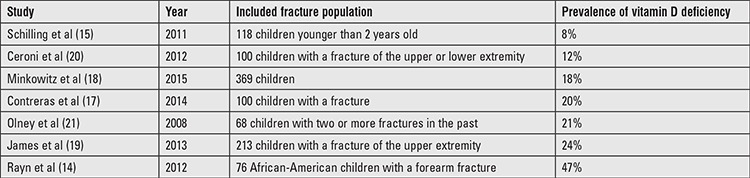
Reported prevalence rates of vitamin D deficiency (calcidiol <50 mol/L) in pediatric fracture patients

**Table 2:**
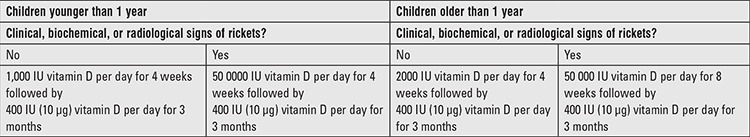
Schedule for supplementation of children with vitamin D deficiency (25-hydroxyvitamin D <50 nmol/L)

**Table 3:**
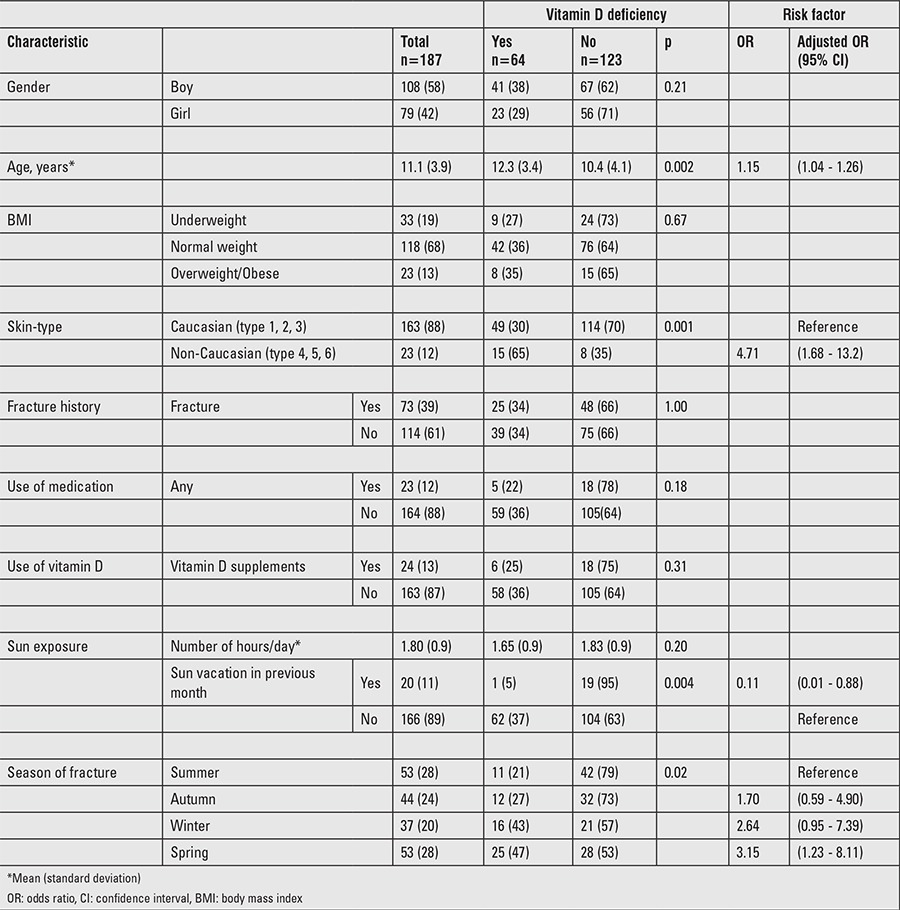
Univariable association and multivariable logistic regression analyses of patient characteristics/risk factors for vitamin D deficiency. Results are presented as numbers (%) unless indicated otherwise
